# Acute Liver Injury From Mushroom Ingestion: A Timely Intervention in Mushroom Poisoning

**DOI:** 10.7759/cureus.45818

**Published:** 2023-09-23

**Authors:** Hameed Ur Rahman, Syed Yasir Shah, Muhammad Asfandiyar Ali, Riazullah, Abu Baker Khan

**Affiliations:** 1 Internal Medicine, Ayub Teaching Hospital, Abbottabad, PAK; 2 Internal Medicine, Khyber Teaching Hospital, Peshawar, PAK; 3 Internal Medicine, Naseer Teaching Hospital, Peshawar, PAK

**Keywords:** amanita phalloides, abdominal pain, toxicity, liver failure, mushroom

## Abstract

Mushroom poisoning, known as mycetism, represents a pressing health concern worldwide. Although the majority of mushroom ingestions are benign, select species like “Amanita phalloides” can induce catastrophic liver damage, culminating in acute liver failure. In this report, we detail a case involving a 35-year-old female who presented to the emergency department exhibiting symptoms of nausea, vomiting, abdominal pain, and palpitations merely six hours post-ingestion of “Amanita phalloides.” Accurate identification of the specific mushroom species consumed proves challenging in over 90% of poisoning incidents, underscoring the necessity for clinical vigilance. While many mushroom exposures lead to mild gastrointestinal symptoms, recognizing the potential for severe outcomes is paramount for timely and effective intervention.

## Introduction

Mycetism, often termed mushroom poisoning (MP), is a significant global health concern. While certain mushroom types are safe for consumption, offering antioxidative and medicinal benefits, there have been cases where mushrooms deemed edible were later found to be toxic [1]. Mushroom toxicity is especially prevalent in rural areas, given the existence of over 5,000 mushroom species worldwide [2]. In over 90% of poisoning incidents, the exact mushroom species consumed remains unidentified due to the difficulties in accurate species recognition. Although the majority of ingested mushrooms are non-toxic or only cause mild to moderate gastrointestinal symptoms [3], it is crucial to discern MP based on clinical manifestations, laboratory tests, and, when possible, mushroom identification. Often, in scenarios where mushroom samples are unavailable, healthcare practitioners, including emergency physicians and clinical toxicologists, must diagnose MP based solely on clinical presentations since consultations with mycologists might not always be possible [4].

Regarding its manifestation, MP is categorized into three groups: early onset (less than six hours after ingestion), which typically affects the nervous and digestive systems and can cause allergic reactions; late onset (6-24 hours post-ingestion) affecting organs like the liver and kidneys and leading to conditions like erythromelalgia; and delayed onset (more than one day post-ingestion) that affects the kidneys and nervous system and can lead to muscle breakdown [4].

## Case presentation

A 35-year-old female patient presented to our emergency department with complaints of nausea, vomiting, abdominal pain, and diarrhea that began approximately six hours after consuming certain “edible” mushrooms and persisted for two days. Upon initial assessment, the patient exhibited deep jaundice and was dehydrated. The patient had a blood pressure reading of 100/70 mmHg and a pulse rate of 96 bpm, respiratory rate of 20 cycles per minute, oxygen saturation of 94% on room air, and temperature of 99 °F. Neurologically, the patient was drowsy, scoring 13/15 on the Glasgow Coma Scale (GCS) on initial assessment in the emergency room but demonstrated normal tone and power in both the upper and lower limbs. Her pupils were equal in size and responsive to light. All her reflexes were intact. Regarding management of the patient in the emergency department a nasogastric tube was passed, and gastric lavage was done with 50 g activated charcoal. As her pain and vomiting lessened after receiving an injection of ondansetron 8 mg and a lactated ringer of 1 liter, she became oriented in time, place, and person. Abdominal examination revealed epigastric tenderness, and striae gravidarum from previous pregnancies. The rest of the physical examinations were unremarkable.

**Figure 1:**
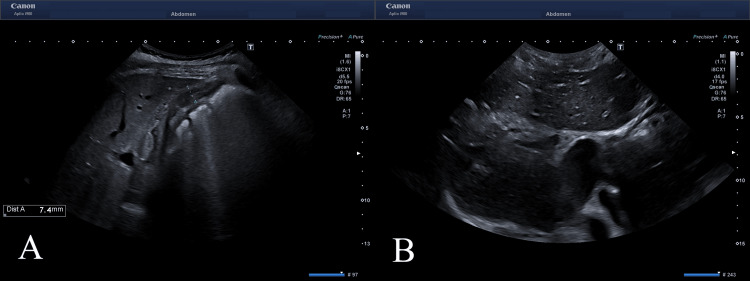
Ultrasound of abdomen. (A) Diffuse gall bladder wall edema along with hypoechoic hepatic parenchyma giving starry sky appearance, suggestive of presence of Hepatitis. (B) An enlarged liver with starry sky appearance.

The patient's past medical history was unremarkable, and she was not taking any over-the-counter (OTC) medicines. Relevant laboratory investigations were conducted based on the patient's clinical presentation. Blood tests showed serum albumin level of 3.3 g/dL (normal range 3.5-5.5 g/dL), markedly elevated levels of alanine transaminase (ALT) and aspartate transaminase (AST), bilirubin, alkaline phosphatase, prothrombin time (PT), and international normalized ratio (INR). The patient arterial blood gas analysis was within normal limits. Screening for viral hepatitis, including Hepatitis A IgM, Hepatitis E IgM, Hepatitis B surface antigen, Hepatitis B core antibody IgM, and anti-hepatitis C antibody, all returned non-reactive. The patient's renal function test showed serum creatinine of 1.66 mg/dL (normal range; 0.7-1.4 mg/dL) and blood urea of 138 mg/dL (normal range; 18-45 mg/dL). An abdominal ultrasound showed diffuse gallbladder wall edema with a collapsed lumen and an enlarged liver with hypoechoic parenchyma giving a starry sky appearance indicative of acute hepatitis (Figures 1A, 1B).

Given her history, clinical presentation, and laboratory findings an INR of 4.31, ALT of 2,520 IU, AST of 2,123 IU, and a total bilirubin of 8.6 mg/dL - a diagnosis of acute liver failure (ALF) was established. Pertinent laboratory values are detailed in Table 1.

**Table 1:** Serial laboratory ivestigations PT: Prothrombin Time, INR: International Normalized Ratio, ALT: Alanine Transaminase, AST: Aspartate Transaminase, TB: Total Bilirubin, PLT: Platelet, HB: Hemoglobin level

Day	PT	INR	ALT	AST	TB	PLT	HB
	Reference value 11-14 seconds	Reference value 0.8-1.1	Reference value 10-50 U/L	Reference value 10-36 U/L	Reference value 0.1-1.0 mg/dL	Reference value 150-450 per microliter	Reference value 12-16 mg/dL
Day zero	56.6	4.31	2520	2123	8.6	161	16.5
Day 1	62.4	4.63	2704	2460	9.4	138	15.7
Day 2	48.7	3.93	2408	2045	7.6	125	15.6
Day 3	28.6	2.61	1860	1620	6.1	130	15.7
Day 4	25.3	2.53	1235	1132	5.3	105	15.7
Day 5	22.4	2.1	931	851	4.7	121	15.6
Day 6	16.3	1.6	681	550	3.9	130	15.7
Day 7	14.2	1.0	430	340	3.2	142	15.5
Day 8	11.1	0.8	285	220	2.5	145	15.8
Day 9	11.1	0.8	195	144	1.7	147	15.8

For management, the patient was administered intravenous fluids, specifically a liter of 0.9% normal saline as a bolus, followed by 5% dextrose saline infusion twice daily. She also received omeprazole 40 mg intravenously daily, and oral Silamyrin 200 mg twice daily. She was given an infusion of penicillin G in doses of 1,000,000 U/kg/day for six days and ornithine, vitamin B2 and B3 supplement. Due to coagulopathy, vitamin K (10 mg) was administered intravenously daily for three days. Given her hyperkalemia, the patient was treated with calcium gluconate 10% (10 mL in normal saline) over 10 minutes, repeated twice over six days. Rifaximin 550 mg was introduced orally twice daily after an initial 48-hour period. Owing to the patient's elevated PT and INR, she received 13 units of fresh frozen plasma over three days to prevent bleeding complications.

Following the supportive treatment regimen, the patient exhibited marked improvement and was subsequently discharged on the ninth day of admission with a prescription for oral multivitamins and liver tonics. On her two-week and four-week follow-up visits, all laboratory markers showed significant improvement.

## Discussion

Approximately 100 mushroom species are recognized as toxic to humans. The worldwide incidence of MP varies considerably. Reported mortality rates fluctuate between 10% and 15%, and in certain cases, can even reach 21.2% [5]. Patient outcomes are notably heterogeneous and depend on multiple determinants including the type of mushroom consumed, the amount ingested, the time elapsed before presenting to the emergency department, and the therapeutic strategies implemented. These manifestations are generally categorized into specific types such as gastrointestinal, hepatic, nephrological, neuropsychiatric, hemolytic, and photoallergic reactions. These classifications are contingent on the particular mushroom species consumed and the toxins they embody [5].

**Figure 2:**
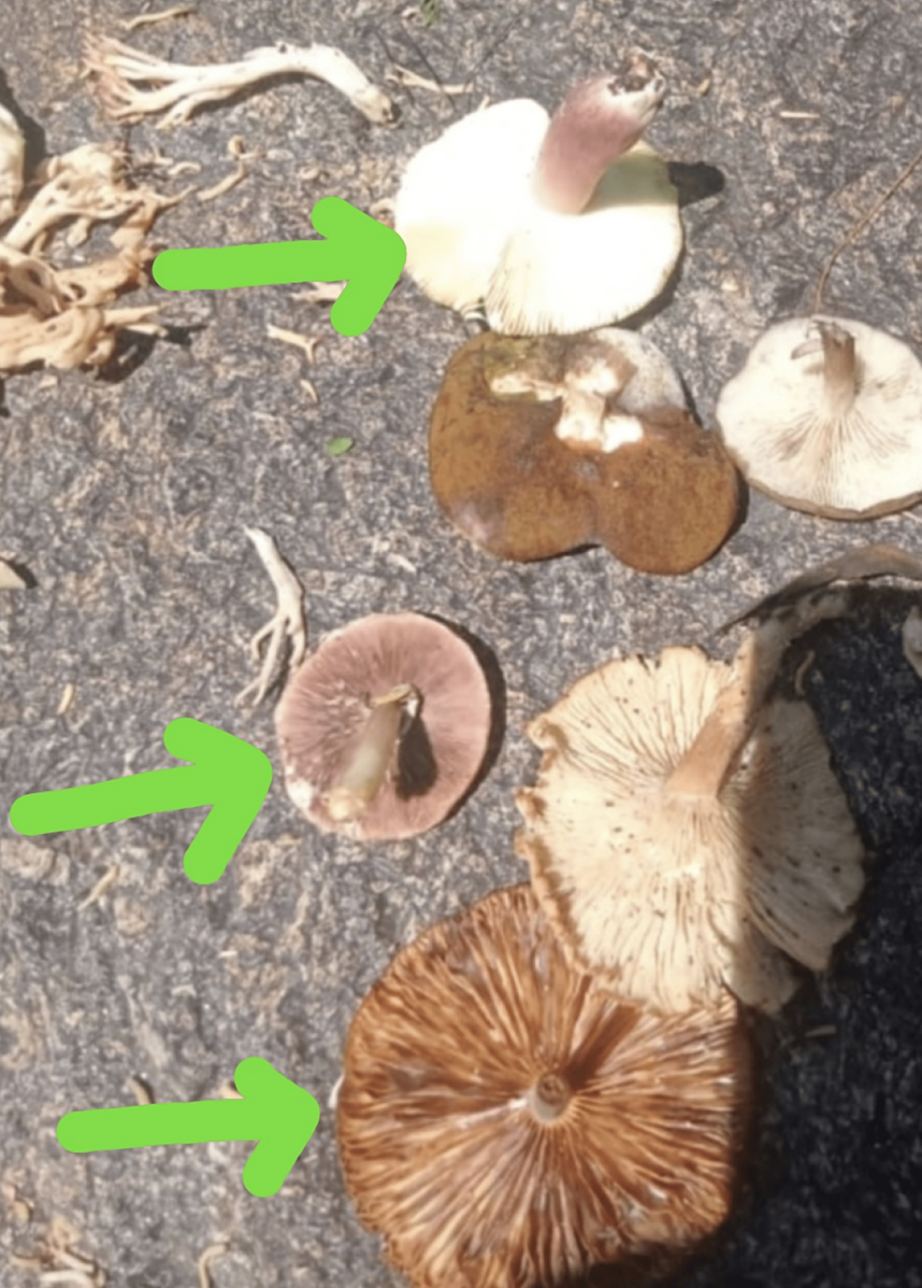
Amanita phalloides are commonly found in the northern regions of Pakistan. This specimen was associated with the acute liver injury case described herein.

In the case under discussion, symptoms manifested approximately six hours post-ingestion, presenting as generalized pallor, sweating, palpitations, and altered liver function tests (LFTs). The causative mushroom was suspected as “Amanita phalloides,” as illustrated in Figure 2 based on the relevance of symptoms and pictures of this class of mushrooms. It is well established that wild mushroom ingestion can detrimentally affect the liver, kidneys, and nervous system. For example, “Conocybe lactea” is known for its hepatotoxic properties due to its phallotoxin content, a toxin notoriously associated with the lethal “Amanita phalloides” mushroom [5].

Two primary mechanisms elucidate the hepatocellular apoptosis induced by amatoxins [6]. Firstly, amatoxins bind non-covalently to RNA polymerase II, inhibiting hepatocyte protein synthesis and RNA polymerase II activity. The liver, with its high protein synthesis and cellular turnover, is particularly susceptible to amatoxin toxicity compared to other organs [7]. A secondary mechanism proposes that amatoxins stimulate cytokine production, particularly tumor necrosis factor-alpha (TNF-α). The combined effect of amatoxin and TNF-α culminates in hepatocyte apoptosis [8]. Additionally, some hypotheses suggest a role for amatoxin-induced free radical generation in hepatocellular damage. Evidence suggests that amatoxin increases superoxide dismutase production while reducing catalase activity, thereby promoting hepatic dysfunction via a peroxidative pathway [9].

Patients with amatoxin poisoning generally exhibit four distinct clinical phases. The latent phase ensues post-ingestion, during which patients remain asymptomatic for six to 12 hours. This is followed by a gastrointestinal phase characterized by abrupt onset of symptoms such as nausea, vomiting, diarrhea, abdominal pain, and electrolyte imbalances. This phase typically persists for 12 to 24 hours. Subsequently, a temporary reprieve, termed the “pseudo-remission period” or the false recovery phase, may occur for 12 to 24 hours. The subsequent and most critical phase involves hepatic impairment, with some patients progressing to ALF, as observed in our case [8].

In terms of management, various therapeutic interventions are employed for amatoxin-induced ALF. Strategies like gastric lavage with activated charcoal administration aim to reduce toxin absorption by inhibiting enterohepatic circulation [10]. Pertaining to our case, the patient was managed with intravenous fluids (normal saline, dextrose saline), antipyretic measures like cool compresses and baths to lower body temperature, hepatoprotective agents, and rifaximin.

## Conclusions

MP can lead to serious or even fatal outcomes, even if overt signs of liver or kidney failure are not present. Among the under-recognized repercussions is the potential for cardiac complications, notably arrhythmias that can manifest as sinus, atrial, or ventricular disturbances. In the case presented, the patient experienced an acute liver injury but responded favorably to timely and supportive treatment. It should be noted that severe cases of MP often necessitate interventions such as blood transfusion or hemodialysis for optimal outcomes. While the lifesaving and definitive measure in fulminant ALF is liver transplant, fortunately, it was not the case here.
